# Ultrasound assessment of SARS-CoV-2 pneumonia: a literature review for the primary care physician

**DOI:** 10.1080/07853890.2022.2067896

**Published:** 2022-04-23

**Authors:** Damiano D’Ardes, Claudio Tana, Alessandro Salzmann, Fabrizio Ricci, Maria Teresa Guagnano, Maria Adele Giamberardino, Francesco Cipollone

**Affiliations:** a “Clinica Medica” Institute, “SS. Annunziata” Hospital of Chieti, Department of Medicine and Aging Sciences, “G. D’Annunzio”, University of Chieti, Pescara, Italy; b “Geriatric and COVID-19 Unit”, “SS. Annunziata" Hospital of Chieti, "G. D'Annunzio" University of Chieti, Pescara, Italy; c Department of Neuroscience, Imaging and Clinical Sciences, "G. d'Annunzio" University, Chieti, Italy

**Keywords:** Novel coronavirus disease, SARS-CoV-2, COVID-19, ultrasound, hospital, primary care, healthcare

## Abstract

The SARS-CoV-2 pandemic is considered one of the most critical global health emergencies in the last century. The diagnostic approach to the novel coronavirus disease (COVID-19) and its possible complications through a point-of-care-ultrasound (POCUS) evaluation could represent a good solution in the primary care setting. POCUS is a non-invasive technique that can be used outside hospitals to screen COVID-19 patients and their complications safely. Moreover, it offers several applications of diagnostic evaluation not only on lung parenchyma but also to search disease complications, such as the cardiovascular system, even at the patients' home. This narrative review aims to analyse the literature and provide data to primary care physicians engaged in monitoring and treating patients with SARS-CoV-2 infection.

**Key Messages**
1. POCUS is an important tool for the diagnostic approach in the primary care setting already before the start of the SARS-CoV-2 pandemic.
2. Portable devices are useful in monitoring the clinical evolution of patients with infection from SARS-CoV-2 at home.
3. The ultrasonographic features can help the general practice physicians to evaluate the presence of lung involvement and to diagnose complications from the SARS-CoV-2 infection involving districts such as the cardiovascular system.

## 1. Introduction

The recent pandemic from the novel coronavirus disease (COVID-19) is considered one of the most critical global health emergencies in the last century [1], being associated with millions of affected cases and deaths worldwide [2]. Healthcare systems have been severely tested, in particular hospitals and primary care settings. The SARS-CoV-2 pandemic imposed a series of changes into diagnostic and treatment approaches because of the risk of virus transmission and the need of working in sterile environments. In this setting, the recent diffusion of handheld ultrasound devices has led to significant clinical benefits. The diagnostic approach to COVID-19 and its possible complications through a point-of-care-ultrasound (POCUS) evaluation could represent a good solution in the primary care setting [3]. The need to evaluate a vast population of patients, combined with the need to reduce the risk of virus transmission for both operators and patients, represents a critical point in all care areas, from hospital units to emergency and primary care settings [4]. The low availability of diagnostic methods is associated with an urgent need to find solutions to achieve prompt diagnosis, fast treatment initiation and effective triage of patients at risk of clinical deterioration who need urgent hospitalisation [5]. In this narrative review, we aimed to examine the role of POCUS during the COVID-19 pandemic and to rethink the usefulness of its long-term use for clinical governance in the primary care setting.

### 1.1. Literature search

We performed a literature search on public databases (Pubmed, Scopus, Cochrane library) from December 2019 through November 2021, focussing on articles relating to the use of POCUS in COVID-19 in the primary care setting in terms of diagnostic, therapeutic strategies and risk stratification algorithms. The use of POCUS to assess COVID-19 pathological findings and complications in adult and paediatric patients were searched.

Search terms were:

- COVID-19 AND primary care AND ultrasound OR bedside ultrasound OR POCUS;
- SARS-CoV-2 AND primary care AND ultrasound OR bedside ultrasound OR POCUS;
- novel coronavirus disease AND primary care AND ultrasound OR bedside ultrasound OR POCUS.

### 1.2. The usefulness of POCUS in the primary care setting

POCUS is a valuable tool in primary care setting, capable of refining diagnostic and therapeutic pathways, therefore contributing to better patient outcomes. However, its integration into the primary care setting requires caution due to the differences between primary care and other treatment settings [6]. Wordsworth [7] analysed its usefulness by comparing costs and quality of scans in a primary care setting *vs* an urban teaching hospital, showing that scanning patients at the primary care level reduce the number of hospital scans, out- and in-patients visits and emergency admissions. Although costs are higher in the outpatient service than the hospitals, the total average costs are lower because of the avoidance of hospital admission [8]. Steinmetz et al. [9] pointed out some of the most valuable and common applications of POCUS in the primary care setting, ranging from screening tools of abdominal aortic aneurysms, intrauterine pregnancy, gallstones, severe left ventricular dysfunction, and diagnosis of musculoskeletal injuries.

Moreover, Andersen et al. [10] have shown an overall positive experience by patients undergoing POCUS in a general physician's (GP's) office: above a total of 691 patients interviewed, 96% of them reported that they had a very positive (*n* = 334) or positive (*n* = 220) experience of being examined with POCUS by the GP. Despite this, there is a large variety in primary care physicians' access to home ultrasound. Andersen et al. [10] showed that in a sample of GP's clinics in 20 European countries, in-house access to abdominal ultrasound was widespread in Germany (98.0%), Slovenia (41.4%) and Switzerland (40.6%), but somewhat less available in Croatia (1.5%) and Denmark (1.9%). Pelvic ultrasound, instead, in comparison with abdominal ultrasound, was less commonly used in general, with the highest percentage of application in Finland (30.8%), POCUS could offer several potential advantages in terms of clinical management and integration into primary care services, even if there are still missing data on diagnostic accuracy and outcomes.

## 2. Pocus in COVID-19: points of interest and strengths

POCUS is a non-invasive technique to screen COVID-19 patients safely, using the correct disinfection protocol of the ultrasound transducer [11]. It is a method of low cost, rapid application and allows the patients to avoid exposure to radiations of other imaging techniques such as computed tomography. Moreover, it offers a wide range of well-known applications that can be beneficial in terms of faster diagnosis [12]. Even if the lung is the most affected organ in COVID-19, myocardial injury and thromboembolism may be present [13]. Therefore, the swift bedside evaluation of the heart, chest and vessels using POCUS has brought this tool to the forefront of the fight against COVID-19 [14]. The Table 1 summarises all the advantages and strengths of using ultrasound in COVID-19.

**Table 1.** Synthesis of all the advantages and strengths of using ultrasound in COVID-19.

**POCUS in COVID-19: points of interest**
Low cost
Rapid
No exposure to radiations
Bedside (home-care)
Lung evaluation
Cardiac evaluation
Vessels evaluation

### 2.1. Diagnosis of SARS-CoV-2 infection and pneumonia

The diagnosis of SARS-CoV-2 infection is reached by nasopharyngeal swab with RT-PCR (reverse-transcriptase polymerase-chain-reaction). This method shows a good accuracy in detecting the presence of SARS-CoV-2 infection, with a relevant sensitivity [15], and it is considered the technique of choice in the diagnosis of SARS-CoV-2 infection [16]. In hospital and emergency care settings, the diagnosis of COVID-19 pneumonia is usually reached by computed tomography (CT), by revealing ground-glass opacities, consolidation, reticular pattern and crazy paving pattern [17,18]. In the primary care setting, the use of POCUS could help the physicians better identify patients at high risk of clinical worsening and find signs suggestive of pneumonia or other complications directly at patients' homes. CT is reserved, after hospital admission, to those cases with moderate to severe symptoms and/or with respiratory failure; the sensitivity of chest CT imaging for COVID-19 was 97% [19]. Asymptomatic and mild-symptomatic patients, without lung failure and that can be managed at home, could benefit more from POCUS, preventing unnecessary hospital admissions since the massive overload of the health services [20]. Recently Di Gioia et al. [21] found that lung ultrasound (LUS) alone in the Emergency Department (ED) has a diagnostic perfomance high enough to play a role in ruling out COVID-19 pneumonia, showing a sensitivity of 85.6%, specificity of 91.7% and overall diagnostic accuracy of 89.4%.

### 2.2. Tool for triage and diagnostic-therapeutic framework

During the COVID-19 outbreak, patients with COVID-19 were managed according to well-defined pathways from primary care services to COVID-19 hospitals. In the COVID-19 Hospitals, Triage or Pre-Triage Units were located outside the Emergency Department, with different levels of organisation, but constituted mainly by dedicated physicians, paramedics and adequate equipment, with the ability to perform rapid tests, RT-PCR tests, serologic testing and ultrasound, since POCUS has been suggested as a tool in the early diagnosis and follow-up of COVID-19 patients [22].

Studies on clinical manifestations of COVID-19 with POCUS imaging include several findings, but most of them are not specific for COVID-19. The BLUE protocol, developed in 2008, allows quick differential of patients presenting in the ED with acute respiratory failure [23]. The pandemic has represented an opportunity to set new algorithms and protocols. In fact, Bianchi et al. [24] applied a 12-scan protocol for COVID-19 patients to stratify their diagnostic process, with 86% sensitivity, 71% specificity and 89% negative predictive value. Moreover, Morin et al. [25] developed an algorithm inspired by Lichtenstein's BLUE protocol with the hypothesis that LUS performed during the initial examination can detect high-risk patients with COVID-19 and address the patients to better management. Narinx et al. [26] confirmed that the application of a 6-scan POCUS protocol in early triage in the Emergency room might provide a practical, rapid, low threshold and safe screening tool in the evaluation of a possible COVID-19 disease, with high sensitivity (93.3%) and negative predictive value (94.1%).

### 2.3. Diagnosis of cardiovascular, thromboembolic complications and ventilation/hemodynamic monitoring

Concerning cardiovascular management, POCUS is widely used to assess the presence of previous heart disease and monitor clinical cardiological developments in the clinical context of COVID-19. The ultrasonographic approach gives the physicians of primary care and COVID-19 hospitals the opportunity to evaluate the cardiovascular system and screen the thromboembolic risk. The latter is known to be significantly increased in COVID-19 due to the endothelial damage, recurring in particular to CUS of lower limbs veins. Both cardiac and vascular ultrasound monitoring could be applied in primary care by experienced GP or specialists, even though existing POCUS guidelines are based mainly on hospitalised patients and may not apply to general practice [27].

The ultrasonographic evaluation could also be precious in monitoring hospitalised patients with COVID-19 as a decision-making tool in ventilatory support and fluid therapy strategies. Moreover, if compared to ARDS, COVID-19 shows some peculiar characteristics that have led to the identification of two phenotypes [28], which can be differentiated on the basis of three parameters: the severity of the infectious disease and the cell-mediated response, the response to ventilation therapy and the time between the onset of symptoms and the need for hospital treatment. This distinction, which seems to be more effectively performed by CT diagnostics [29], can be alternatively made by LUS [30], identifying in particular two phenotypes: the L-type, characterised by a reduced lung elastance with consequential high compliance, and the H-type, which on the other hand shows low lung compliance, opposed to high elastance. The differences between the two phenotypes affect the type of ventilation that must be applied. Specifically, the L-type appear to be more responsive to low levels of PEEP, while the H-type, which is closer to a classic form of ARDS, would better respond to high PEEP values therapy. Thus, it seems clear that the role of POCUS and, more specifically of LUS, is crucial, as underlined by Hussain [4] according to these lines of recommendation in respiratory failure due to COVID-19:

1. multiorgan POCUS and LUS show a maximum efficacy compared to any other imaging technique available in the guide of adequate ventilatory support;
2. LUS is considered more effective than X-ray and on au pair with CT to provide a guide to the appropriate clinical approach;
3. multiorgan POCUS shows greater efficacy than LUS alone in guiding the correct clinical approach.

Moreover, regarding the application of POCUS to fluid therapy management, the ultrasonographic evaluation has yet been widely used before the pandemic in the critically ill patient (e.g. VExUS score [30]) and has mainly been used during COVID-19 pandemic also in patients treated with primary care services, through the assessment of the degree of collapse and/or distensibility of inferior cava vein and also through the evaluation of a B-line pattern.

## 3. Pocus in COVID-19: thoracic investigation

### 3.1. Pocus in COVID-19: pulmonary investigation

The lower airways represent the main target of infection for the SARS-CoV-2 virus. The study of the lung parenchyma through CT examination typically shows bilateral interstitial pneumonia with irregular asymmetrical lesions, mainly distributed in the peripheral portion [29]. This distribution allows the execution of an effective ultrasound examination. If physicians with good experience perform this exam could identify the degree of severity of the disease and consequently can be helpful to manage the patient towards the appropriate clinical setting.

**Figure 1.**
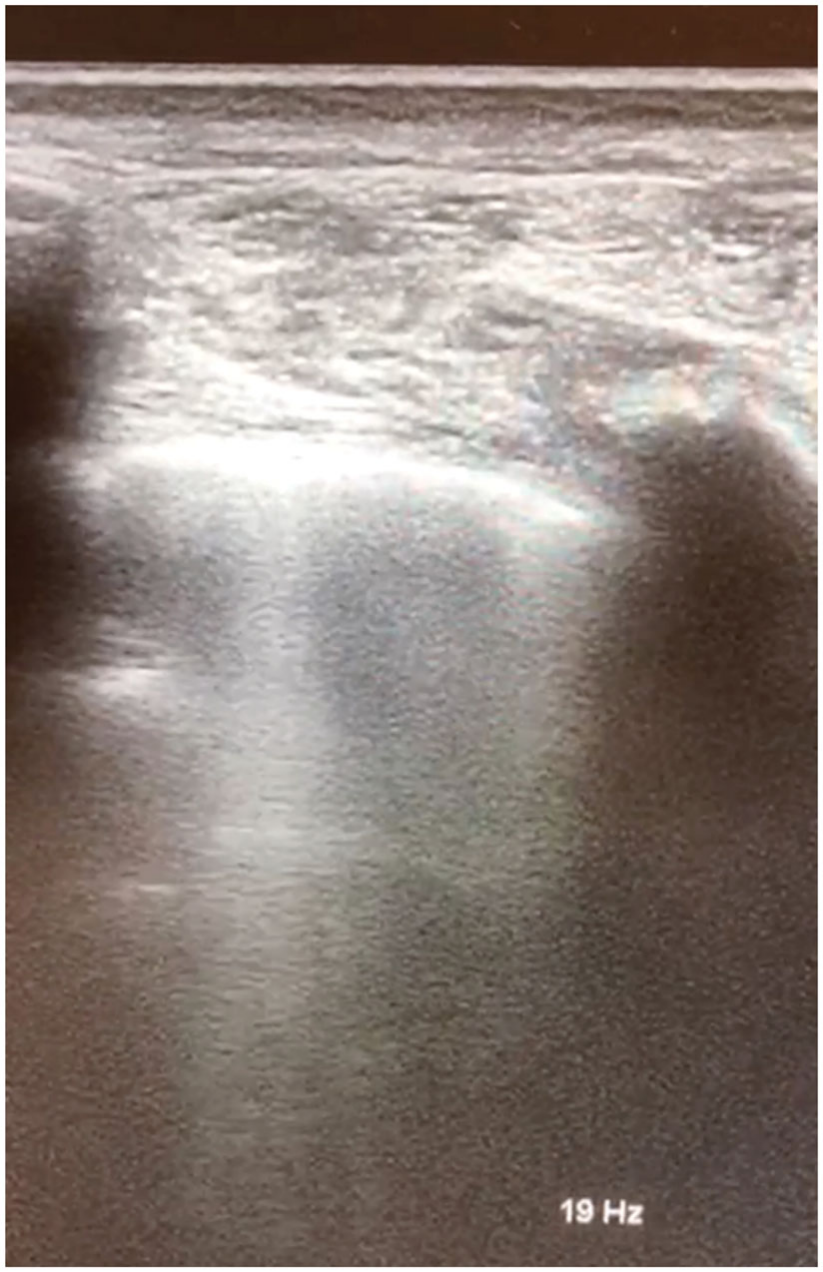
Bedside evaluation by a primary care physician of a patient with SARS-CoV-2 infection: B-lines at lung ultrasound appear as slightly hyperechoic bundles perpendicular to the hyperechoic pleural line.

The typical ultrasound features of lung involvement from COVID-19 [31,32] are the presence of B-lines (Figure 1) organised in bilateral multiform clusters alternating with savings areas, which sometimes have the appearance of a white lung. More specifically, the authors have defined a “light beam” indicating with this term a vertical, lucent and band-shaped artefact that moves with sliding, at times creating an "on-off" artefact that appears and disappears from the image. It could be explained as a feature of an initial phase of the disease, but it is observable also in other pathological conditions [33]. However, if it is related to a suggestive clinical picture of SARS-CoV-2 infection, this ultrasonographic feature seems beneficial because it could help primary care physicians evaluate the presence of lung involvement.

**Figure 2.**
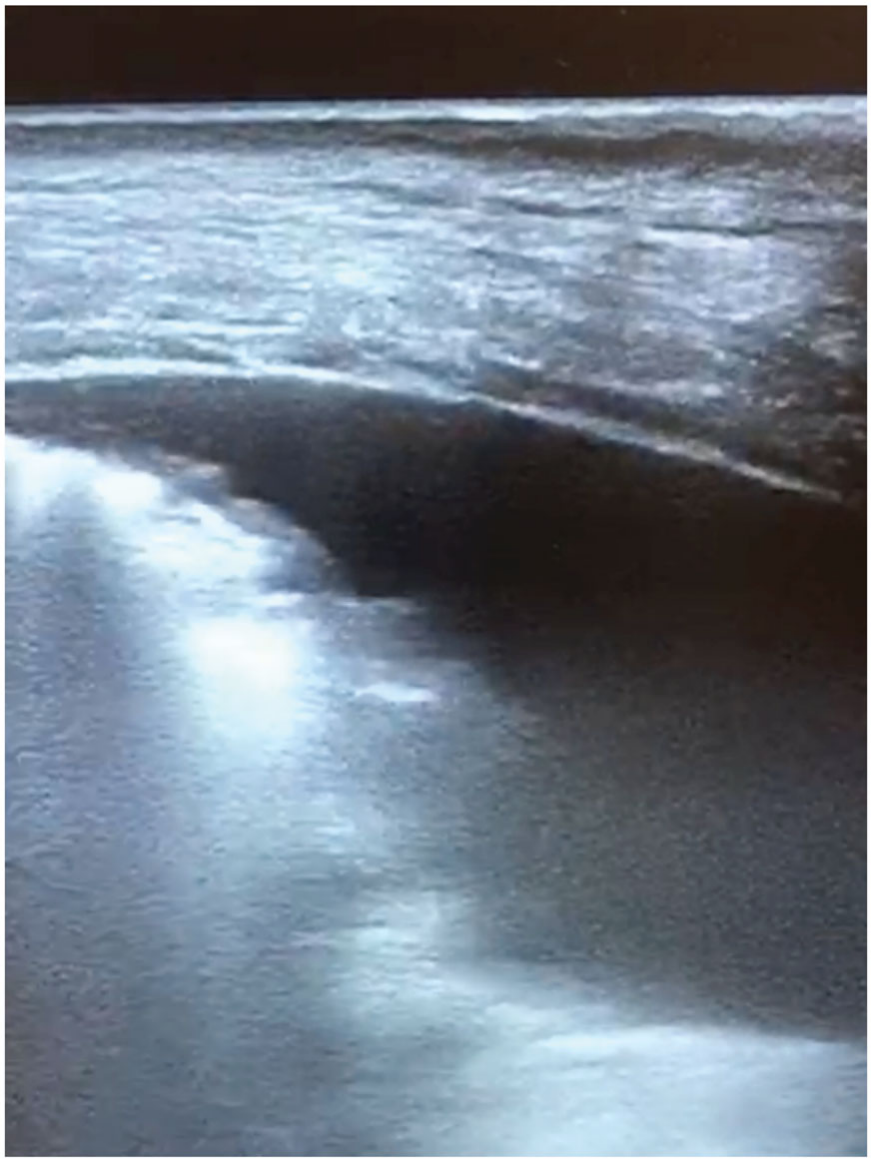
Pleural effusion at lung ultrasound in a SARS-CoV-2 patient appears as an anechoic area (on the right region of the picture).

Moreover, the physicians could also find consolidations, suspected signs of bacterial superinfection and the presence of complications as pleural effusion, more frequently present in patients with a suggestive history of cardiovascular disease (Figure 2).

Some imaging scores have been used to define the extension of lung damage. The LUS score is a semiquantitative parameter of the severity of pulmonary impairment, and it is based on the presence of alterations such as B-lines, pleural line abnormalities and lung consolidations. Lichter et al. [34] have found that pathological LUS patterns, for a total LUS score >18, were associated with extensive damage, clinical deterioration, and, consequently, the need for non-invasive mechanic ventilation. The prediction accuracy for this cut-off is low (sensitivity = 62%) instead of a cut-off of 20, which seems to have more accuracy in predicting the mortality from COVID-19 pneumonia [35].

In this setting, it is important to compare LUS and traditional radiology imaging, in particular chest X-ray and lung CT scan, in the diagnosis of COVID-19 pneumonia and management of COVID-19 patients (Table 2). As mentioned before, LUS has several advantages as an easy bedside use, even in patients in ICU, with the possibility of a diagnosis at home patients. Moreover ultrasound does not involve the use of radiations. It has also been showed that LUS has been associated with severity of P/F ratio and with the extent of the inflammatory response [36].

**Table 2.** Comparison of LUS and CT scan imaging in the assessment of COVID-19 patients.

	LUS	CT imaging
PRO	Good diagnostic performance Bedside evaluation Diagnosis of COVID-19 pneumonia at patient’s home Screening of complications (e.g. dehydration, heart involvement, etc.) Early screening of patients to refer to the hospital admission	High diagnostic accuracy High reproducibility Standardised protocols
CONS	Adequate training and experience Operator dependence No standardised protocols	Exposition to radiations Not available at patient’s home

However, LUS is operator-dependent and clinicians should receive a specific training and there is not a standardised protocol for LUS. CT imaging provides instead a high diagnostic accuracy even if the use of radiations limit its use in some populations such as paediatrics and it is not a bedside technique for the diagnosis at patients’ home. A recent meta-analysis has demonstrated a significant higher diagnostic accuracy of LUS versus traditional chest X-ray (86.4% (95%CI: 72.7–93.9) vs 80.6% (95%CI: 69.1–88.6), respectively), confirming the importance of LUS for the diagnosis of COVID-19 pneumonia. Among all techniques, however, chest CT demonstrates the highest specificity to detect lung damage from the SARS-CoV-2 infection (80.0% (95%CI: 74.9–84.3) [37,38].

Another point which has confirmed the importance of LUS in the assessment of COVID-19 pneumonia is the demonstration that LUS correlates well with the visual scoring at CT (*r* = 0.65, *p* < .001) [39]. Moreover, LUS has confirmed its importance also in the ICU department. Chest CT score seems to correlate positively with LUS findings at ICU admission (*r* = 0.953, *p* < .0001) [40].

### 3.2. Pocus in COVID-19: cardiac investigation

Cardiac involvement in COVID-19 disease has been evaluated since the early days of the pandemic, with a wide area of interest involving:

1. patients with pre-existing cardiovascular diseases who have a predisposition to a higher risk of death and worse outcomes if affected by COVID-19, for example, heart failure and atrial fibrillation [20,21];
2. patients with COVID-19 showing a tendency to develop cardiovascular complications;
3. some drugs tested in the treatment of the infection that could have cardiovascular side effects;
4. during the pandemic, great interest has been raised regarding the role and safety of angiotensin-converting enzyme (ACE) inhibitors and sartans in correlation with COVID-19 [41]. Echocardiography can document some findings that are useful from a prognostic point of view. Pagnesi et al. compared 200 COVID-19 patients with versus without pulmonary hypertension (PH) and with versus without right ventricular dysfunction (RVD). The prevalence of PH and RVD was 12.0% (24/200) and 14.5% (29/200), respectively. PH was associated with an increased risk of in-hospital death or ICU admission (41.7 vs 8.5% of patients without PH, *p* < .001), meanwhile RVD was not (17.2 vs 11.7% of patients without RVD, *p* = .404) [42].

COVID-19 patients tend to develop pulmonary hypertension associated with hypoxic vasoconstriction of the pulmonary bed, even in the early stages of the disease. In a prospective study of 1216 COVID-19 individuals [43], 667 (55%) patients had alterations at echocardiogram. In particular, left and right ventricular alterations were noted in 479 (39%) and 397 (33%) patients. In patients without a history of cardiac disease (*n* = 901), the echocardiogram was abnormal in 46%, and 13% had cardiac disease with high-grade severity. Independent predictors of ventricular abnormalities included high natriuretic peptides, cardiac troponin and severity of COVID-19 symptoms. The echocardiographic exam changed patient management in 33% of cases. In fact, the echocardiographic data changed the management of disease-specific therapy in 42% of cases (171/405) in terms of therapy for heart failure, acute coronary syndrome, pulmonary embolism or tamponade, and initiating antibiotics in the presence of endocarditis. The echocardiographic data also changed the level of patient care by 8% (32/405) and guided titration of haemodynamic support in 13% (51/405).

All these changes in terms of patient management were reported more frequently in patients with a history of cardiac disease (38% vs 32% compared with those without pre-existing cardiac disease; *p* = .005) and in patients who had elevated cardiac biomarkers compared with patients without these laboratory findings (39% vs 31%, *p* < .001).

In conclusion, cardiac POCUS is a valuable tool to evaluate patients with COVID-19 and suspected cardiac impairment. Also, an operator with a standard level of expertise can early recognise the most important signs of cardiac damage and provide a hospital admission, in particular for those subjects who have a pre-existing cardiac disease and advanced age [44].

## 4. Pocus and COVID-19 in primary care and home monitoring

A debated topic during the pandemic was the use of POCUS in the evaluation of pulmonary involvement from COVID-19 in mildly symptomatic patients at home isolation. In Italy, this monitoring was carried out by physicians called "USCA", that is an acronym for Special Continuity Care Units [45,46], but it was not exclusively an Italian phenomenon, as in France, for example, there was the COVISAN experiment consisting in mobile units that provide COVID-19 home testing, preventative measures and help to isolate positive cases [47]. The French patients were also monitored at home through digital telemedicine systems as COVIDOM [48]. In any case, the pandemic changed the workload for primary care physicians, as in Spain [49].

**Figure 3.**
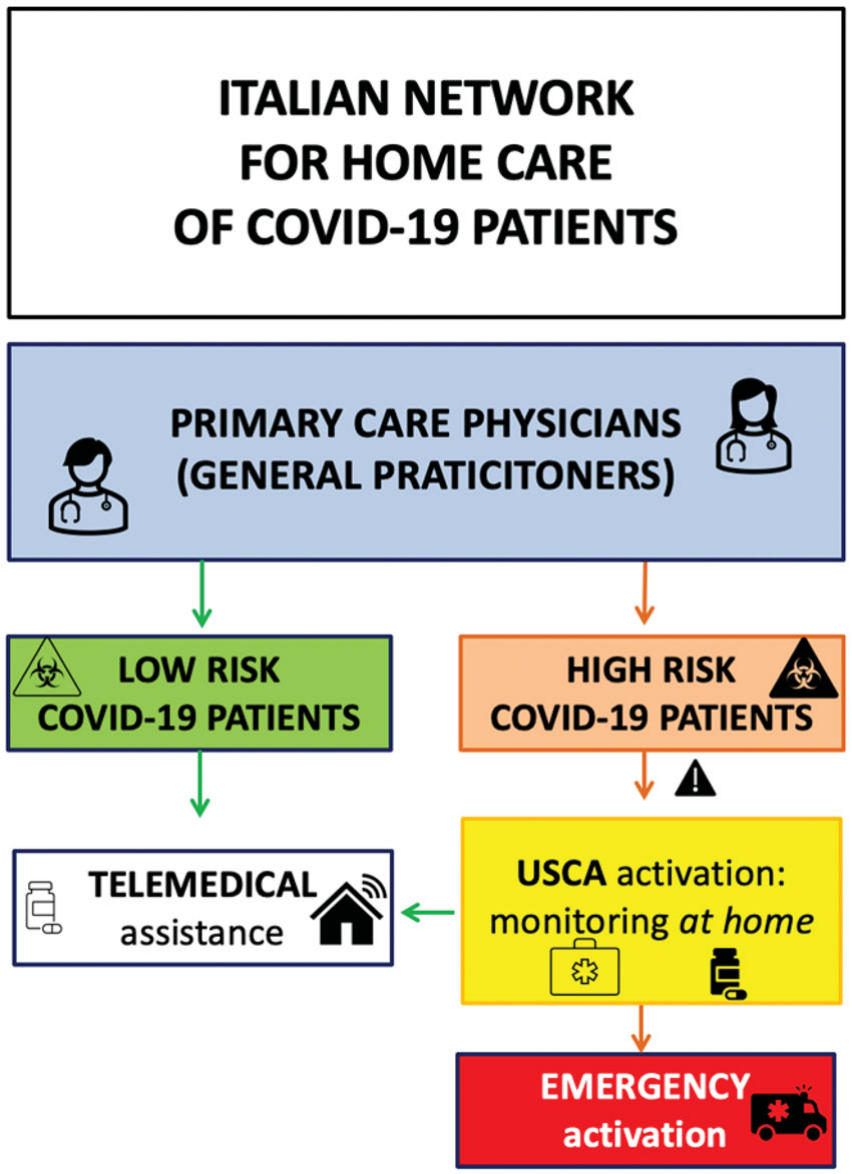
The Italian network between primary care physicians and USCA doctors, who are activated by general practitioners to evaluate at home COVID-19 patients. The USCA doctors in presence of high risk factors, signs suggestive of respiratory failure and bilateral pneumonia could activate emergency system to require hospitalisation for COVID-19 patients in HUB dedicated medical centres.

Focussing on Italian experience, the USCA doctors were activated from primary care physicians to specifically evaluate at home the COVID-19 patients (see Figure 3). Physicians of USCA have been equipped with portable ultrasound scanners and have been trained to execute LUS and to evaluate the presence of the principal complications (as pleural effusions, consolidations, cardiovascular alterations, etc.). They also were taught to monitor the fluid status through the study of inferior cava vein diameter and collapsibility.

The need to use POCUS at home, also thanks to the born of these home health units, has been largely promoted, for obvious technical needs, with a consequent widespread diffusion of portable ultrasound devices. The accuracy of portable devices was a hot topic even before the pandemic [50–52]. They have been indicated before for the study of abdominal organs, trauma but also for the execution of procedures, for example, knee injection for arthritis, finding of ascites or pleural effusion and consequently to follow paracentesis and thoracentesis. Although portable devices have shown good efficacy and reliability, they have some limitations, such as the lack of sensitivity of Doppler US for the evaluation of some superficial structures, vessels, diagnosis of tumours, ectopic pregnancies and the study of the obese patients [53,54].

**Figure 4.**
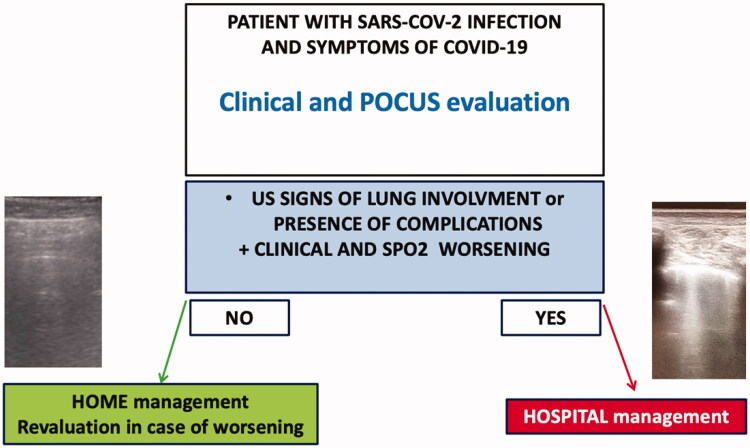
Management of patients with SARS-CoV-2 in primary care. The presence of ultrasonographic signs of lung involvements or the presence of complications (for example, pleural effusions and cardiovascular alterations) in addition to clinical and SpO2 worsening could suggest the physicians to in-hospital management of patients.

The use of POCUS has demonstrated a precious role in monitoring the possible clinical evolution of patients [55]. All the ultrasonographic pathologic features of LUS seem to be helpful in the management of the SARS-CoV-2 patients in the primary care setting, helping the physicians of primary care to evaluate the presence of lung involvement in SARS-CoV-2 infection, together with a clinical and parameters evaluation and guide the clinicians to manage the patients at home or at the hospital as schematically represented in Figure 4. The POCUS evaluation could also represent a tool to diagnose other complications, as pleural effusion or cardiovascular alterations (deep vein thrombosis, cardiac effusion, right ventricle dysfunction, etc.). Thus, the doctors are expected to a global evaluation of COVID-19 patients, directly at home, from the moment they can evaluate SpO2, respiratory frequency, the presence of dyspnoea or other symptoms and the severity of pneumonia through LUS and the appearance of the principal complications, reducing the occurrence of inappropriate access to Emergency services and anticipating the need for hospitalisation in patients who show signs of worsening.

Even in the setting of paediatrics, the ultrasound evaluation of the paediatric patient with COVID-19 has played an essential role since the beginning of the pandemic. In childhood and in the paediatric population, SARS-CoV-2 infection generally produces mild symptoms, though in a small percentage of cases, it can cause severe disease with the need for admission to intensive care and, on rare occasions, can give a fatal outcome [56]. POCUS could help the primary care paediatricians to evaluate paediatric patients with SARS-Co-V-2 infection without recurring to traditional radiology with X-ray and CT [57,58], with an important saving of exposure to radiation, that is a crucial point, especially in children [59]. Recent studies have underlined the importance of LUS in paediatric patients with COVID-19. Hizal et al. [60] showed that LUS confirmed the diagnosis of pulmonary involvement in 83,3% of paediatric patients that had positive CT findings. Moreover, LUS demonstrated normal lung patterns among 93,75% of patients who had normal CT features. The sensitivity and the area under the receiver operating characteristics curve identified by the chest X-ray and LUS tests were compared and significantly different, with superiority of LUS.

Hand-held ultrasound is playing a key role in the diagnosis and management of patients COVID-19 due to its versatility, availability and its inherent advantages in infection control and staff safety [61–63]. Rapid protocols for focussed study generating sufficient information to guide optimum, safe and efficient management have been proposed. POCUS training should be therefore encouraged and further effectiveness studies are eagerly awaited.

## 5. Challenges and future directions

POCUS has been considered an important tool for the diagnostic approach in the primary care setting already before the start of the SARS-CoV-2 pandemic, which has acted as a trigger for an already existing trend, managing to breakdown some methodological and practical resistances and spreading ultrasound clinical investigation [64].

Moreover, the strong diffusion of portable ultrasound equipment has opened new horizons, in particular in terms of primary care health services at patients' homes and to manage special populations, as pregnant women [65].

Before pandemics, authors had already shown the diagnostic importance and usefulness of ultrasound in general medicine, showing that POCUS scans were reported to have a higher diagnostic accuracy, indicating that POCUS could reduce health care costs, even if there were still some uncertainties about the quality of ultrasound portable scans and about training [27]. It is also important to underline the limits of ultrasound technique: although the sensitivity of thoracic ultrasound is high, its specificity may not be the same [66].

The pandemic from COVID-19 is offering new perspectives and resources, regarding challenges and opportunities deriving from new technology. For example, promising studies have shown that nanotechnologies medicine could be applied to fight the ongoing pandemic [67]. Taking this future scenario as an example, we hope that new technologies can also be applied in ultrasound to make diagnostic capabilities even simpler and safer but also to open up the possibility of therapeutic perspectives, which are still not feasible to date, even if some interesting data are emerging [68].

## 6. Conclusions

The use of POCUS through portable devices has shown to have a role in monitoring the possible clinical evolution of patients with infection from SARS-CoV-2. The ultrasonographic features are helpful to manage SARS-CoV-2 patients in the primary care setting and can help the physicians to evaluate the presence of lung involvement and to diagnose complications from the SARS-CoV-2 infection involving the cardiovascular system. The use of ultrasound in primary care is establishing itself and opens up new scenarios with perspectives also thanks to the possible application of emerging technologies.
